# Race/Ethnicity as a Risk Factor in the Development of Postpartum Hemorrhage: A Thorough Systematic Review of Disparity in the Relationship Between Pregnancy and the Rate of Postpartum Hemorrhage

**DOI:** 10.7759/cureus.26460

**Published:** 2022-06-30

**Authors:** Oluwasemilore Okunlola, Shafaat Raza, Stephen Osasan, Sudiksha Sethia, Tayyaba Batool, Zarna Bambhroliya, Joel Sandrugu, Michael Lowe, Pousette Hamid

**Affiliations:** 1 Research, California Institute of Behavioral Neurosciences & Psychology, Fairfield, USA; 2 Neurology, California Institute of Behavioral Neurosciences & Psychology, Fairfield, USA

**Keywords:** pregnancies, childbearing, pregnancy, nationality, ethnicity, race, bleeding, hemorrhage, post-partum hemorrhage, pph

## Abstract

Postpartum hemorrhage (PPH) is a major cause of maternal death and morbidity worldwide. Throughout the years, there have not been many studies looking into the association of race and ethnicity with the occurrence of PPH. The goal of this study was to assess race and ethnicity as risk factors in the development of PPH in pregnant women. Following the Preferred Reporting Items for Systematic Review and Meta-Analyses (PRISMA) standards, we conducted the analysis and conducted a literature search using Google Scholar and PubMed. After applying our inclusion and exclusion criteria, the search technique yielded a total of eight articles. The analysis included seven observational studies and one randomized controlled trial. The incidence of PPH was chosen as the major outcome measure. An evaluation of eight studies revealed that although Hispanics, Asians, Native Hawaiians, and other Pacific Islanders (NHOPI) have a higher chance of developing PPH caused by uterine atony, Caucasians had a greater rate of transfusion than the other groups. In addition, compared to Caucasians, African Americans or African descendants had a lower risk of atonic PPH but increased odds of atonic PPH requiring interventions. On the other hand, compared to non-native groups, Native Americans had increased odds of uterine atony. The results showed that, in contrast to other races/ethnicities, Caucasians had the lowest risk of PPH. Additionally, it was shown that African Americans or those descended from Africans had a higher chance of PPH but a lower risk of atonic PPH.

## Introduction and background

‘‘Maternal mortality health is a very sensitive indicator. All you need to look at is a country's maternal mortality rate. That is a surrogate for whether the country's health system is functioning. If it works for women, I'm sure it will work for men.’’

-Margaret Chan

With a mortality rate of 140,000 yearly, or one maternal death every 4 minutes, postpartum hemorrhage (PPH) is a leading cause of maternal death and morbidity around the world, especially in underdeveloped nations [1]. PPH impacts 5% of all the deliveries, and the majority of deaths occur within 4 hours of delivery, indicating that it is precipitated by the third stage of labor [2,3]. According to the World Health Organization (WHO), in low-income nations, PPH is the leading cause of maternal death, accounting for almost a quarter of all maternal deaths globally [4].

Regardless of the delivery route, hemorrhage greater than or equal to 1,000 mL or hemorrhage followed by clinical manifestations of hypovolemia within 24 hours after the labor and delivery (including intrapartum loss) is defined as PPH by the American College of Obstetricians and Gynecologists (ACOG) reVITALize program. A 10% decrease in hematocrit was previously proposed as a substitute indicator to define PPH; notwithstanding, hemoglobin or hematocrit concentration determinations are regularly delayed, may not accurately reflect the current hematologic status, and thus are not efficacious in the setting of acute PPH [5,6]. The majority of PPH-related deaths happen within the first 24 hours. Most of these difficulties may be avoided if preventive uterotonics are used during the third stage of labor, as well as prompt and adequate treatment [4]. Primary/immediate/early PPH occurs within 24 hours of delivery, while secondary/delayed/late PPH occurs more than 24 hours after birth and up to 12 weeks after birth. PPH can also be classified as the third or fourth stage, depending on if it develops before or after the delivery of the placenta [7,8]. Therefore, the most prevalent etiologies are classified as primary or secondary causes in Table 1 [6,9].

**Table 1:** Etiologies of PPH *Coagulopathies encompass both hereditary and acute anomalies resulting from events like an amniotic fluid embolism. PPH, postpartum hemorrhage.

Primary PPH	Secondary PPH
Uterine atony	Subinvolution of the placental site
Trauma (genital tract laceration)	Retained products of conception
Retained placenta tissue	Infection
Abnormally adherent placenta	Inherited coagulation defects (e.g., factor deficiency such as von Willebrand)
Coagulopathy*	
Uterine inversion	

There are substantial racial and ethnic disparities in pregnancy-related morbidity and mortality, as per various studies [10,11]. However, there has not been much research into the link between ethnicity and the risk of PPH. We may be better able to identify which women are at risk for PPH if we better grasp well-defined independent risk variables. This is noteworthy because obstetrical bleeding, particularly PPH around the world, is a primary cause of maternal morbidity and mortality. Therefore, this study aims to identify the role that race and ethnicity play as a risk for PPH in women of child-bearing age.

## Review

Methods

The Preferred Reporting Items for Systematic Reviews and Meta-Analyses (PRISMA) standards were followed for conducting this systematic review [12]. Relevant articles were found using PubMed and Google scholar. A predetermined set of inclusion and exclusion criteria, as well as a predetermined research topic, guided the study.

Criteria for Incorporation

On February 18, 2022, the most recent search was conducted. Combinations of the following search phrases were used: “pregnancy”, “postpartum hemorrhage”, “in Caucasians”, “in Africans”, “in Hispanics”, “in the Middle East”, “in Spain”, “in Asians”, “in Black Americans”, “in American Indian”. On PubMed, an advanced search strategy was used: “((pregnancy) AND (postpartum hemorrhage)) AND (in Caucasian)”, “((pregnancy) AND (postpartum hemorrhage)) AND (in Africans)”, “((pregnancy) AND (postpartum hemorrhage)) AND (in Hispanic)”, “((pregnancy) AND (postpartum hemorrhage)) AND (in the Middle East)”, “((pregnancy) AND (postpartum hemorrhage)) AND (in Spain)”, ” ((pregnancy) AND (postpartum hemorrhage)) AND (in Asians)”, ” ((pregnancy) AND (postpartum hemorrhage)) AND (in black Americans)”, “((pregnancy) AND (postpartum hemorrhage)) AND (in American Indian)”. Only human studies published between 2012 and 2022, in the English language, were included.

Exclusion Criteria

Exclusion criteria included 1) irrelevant studies, 2) studies published more than 10 years ago, 3) studies not written in the English language, 4) studies with alternative outcomes, and 5) studies not ethnically/racially inclined. 

Primary Outcome Measures

To assess the role of race and ethnicity as risk factors for PPH in child-bearing women.

Data Extraction and Study Variables

Using a specified search method, 286 articles were screened using titles and abstracts after which 275 were excluded. Subsequently, 11 articles were screened by free-full-text articles. Ten articles were screened for eligibility based on the criteria previously mentioned. Data extraction was performed by two independents. The following variables were explored: study design, the purpose of study, sample size, primary endpoints, average maternal age, and time frame data collected.

Results

**Figure 1:**
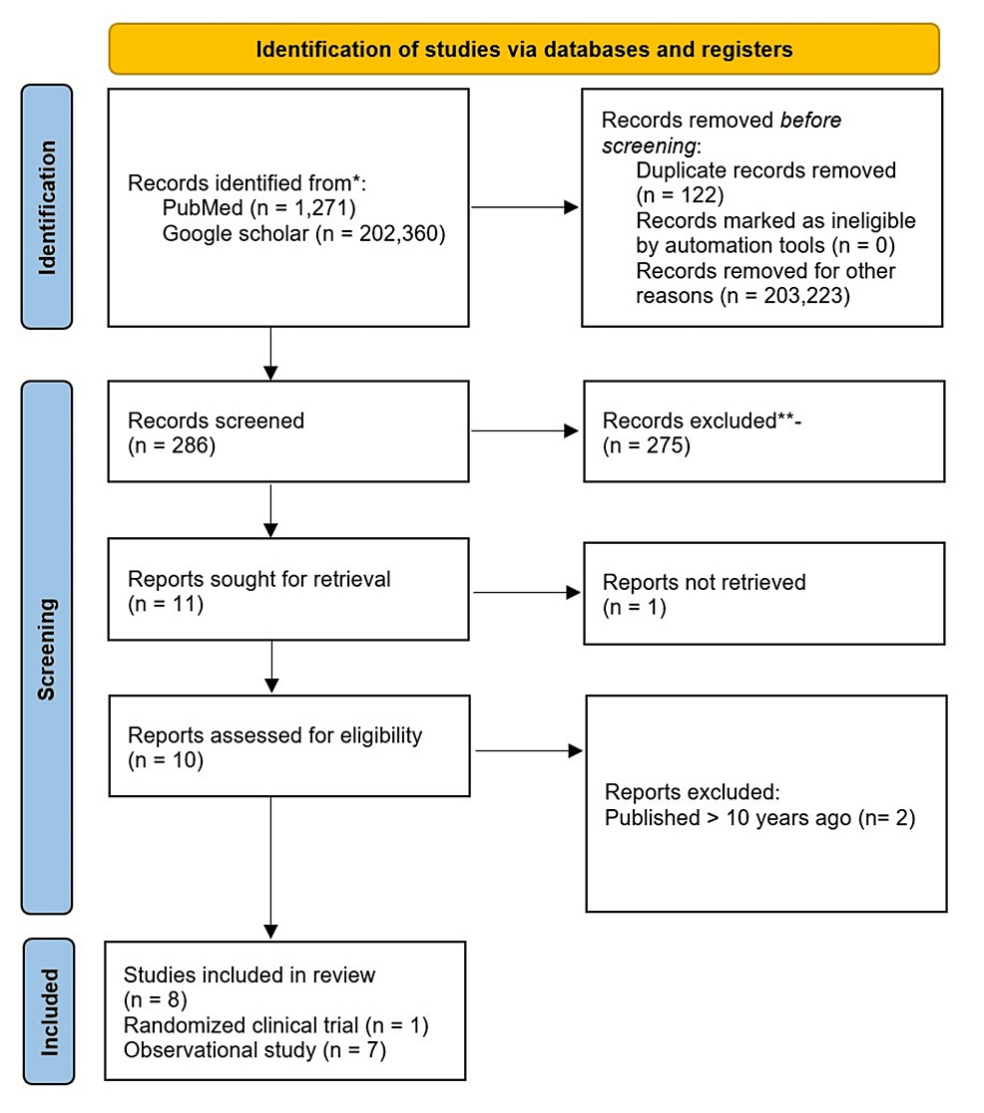
Prisma Flow Diagram PRISMA, Preferred Reporting Items for Systematic Reviews and Meta-Analyses [12].

The following are the results of our search strategy: 1,271 studies were found on PubMed using advanced search words without filters and a total of 202,360 studies were discovered on Google scholar without filters. The search strategy yielded a total of 203,631 studies. Ten full-text publications were possibly acceptable after removing duplications and screening. A total of eight articles were relevant to our study question following the application of inclusion and exclusion criteria. Figure 1 depicts the PRISMA flow diagram for the selection procedure, and Table 2 contains the characteristics of the included studies.

**Table 2:** Baseline Characteristics of the Included Studies ICD, The International Classification of Diseases; RCT, randomized clinical trial; PPH, postpartum hemorrhage.

Author	Year	Country	Study Design		Purpose	Sample Size	Primary Endpoints	Data Collection Time Frame
Bryant et al. [13]	2012	USA		Retrospective cohort study	Examine the impact of race and ethnicity on the risk of atonic PPH, considering potential mediating factors.	2,488,974	(1) Atonic PPH, (2) atonic PPH necessitating transfusion of packed red blood cells, and (3) atonic PPH necessitating hysterectomy.	2005-2008
Harvey et al. [5]	2017	USA		Retrospective cohort study	Examine racial-ethnic variations in the frequency of PPH among Whites, Asians, Native Hawaiians, and Other Pacific Islanders (NHOPI)	243,693	PPH diagnosis at discharge, as defined by ICD-9 codes of 666.x and 641.11.	1995-2013
Kodan et al. [9]	2020	Suriname		Retrospective cohort study	(1) Find out how prevalent PPH is, (2) identify risk factors and root causes of PPH, and (3) assess the management of severe PPH by conducting a criteria-based assessment	9,071	A blood loss of at least 500 mL within 24 hours after the delivery was characterized as PPH.	January 1st - December 31st, 2017
Briley et al. [14]	2014	UK		Prospective cohort study	To quantify reporting inaccuracies, measure the incidence of PPH and establish risk variables for PPH (500 mL) and progression to severe PPH (1,500 mL)	10,213	PPH incidence and risk factors, as well as the progression of PPH to severe PPH.	August 1st, 2008-31st July, 2009
Chalouhi et al. [15]	2015	USA		Retrospective cohort study	To explore if Native American women had a higher risk of PPH following vaginal delivery.	1,062	A visually estimated blood loss of more than 500 mL was categorized as PPH.	June 1st, 2009 -June 30th, 2012
Wetta et al. [16]	2013	USA		RCT	To determine the risk of uterine atony or bleeding.	1,798	Uterine atony or hemorrhage needing treatment.	Not indicated
Reime et al. [17]	2012	Germany		Retrospective cohort study	To determine if there is a link between a mother's origins and a serious disease that puts her on the brink of dying (near-miss).	441,199	The incidences of hysterectomy, hemorrhage, eclampsia, and sepsis	2001-2007
Thepampan et al. [18]	2021	Thai Myanmar		Nested case-control study	Investigate PPH risk factors in pregnant women who had a transvaginal delivery in Northern Thailand in a community hospital	4,845	Within the first 24 hours following delivery, between 500 and 1,000 mL of blood loss	2014-2018

Quality Appraisal of the Trials

Two reviewers (O.O. and M.L.) independently screened the publications using the Newcastle-Ottawa Scale and the Cochrane risk-of-bias instrument to assess the probability of bias in observational research and clinical trials, respectively. Disagreements were resolved by debate until a compromise was achieved. Due to the low variety of research found, the evaluation protected all papers. See Table 3 for a more detailed assessment of the risk of bias in the included studies.

**Table 3:** Quality Assessment of the Included Studies RCT, randomized clinical trial.

Study	Type	Quality Assessment Tool Used	Summary Appraisal-Quality Level
Bryant et al. 2012 [13]	Retrospective case-control	Newcastle Ottawa	High quality (low risk)
Briley et al. 2014 [14]	Prospective case-control	Newcastle Ottawa	High quality (low risk)
Chalouhi et al. 2015 [15]	Retrospective case-control	Newcastle Ottawa	High quality (low risk)
Harvey et al. 2017 [5]	Retrospective case-control	Newcastle Ottawa	High quality (low risk)
Kodan et al. 2020 [9]	Retrospective case-control	Newcastle Ottawa	Some concerns
Wetta et al. 2013 [16]	Secondary analysis of RCT	Cochrane risk of bias	High quality (low risk)
Reime et al. 2012 [17]	Retrospective cohort	Newcastle Ottawa	High quality (low risk)
Thepampan et al. 2021 [18]	Retrospective case-control	Newcastle Ottawa	Some concerns

Baseline Characteristics of the Included Studies

Table 2 lists the features of the studies that were included. One of the eight studies considered in our research was a randomized controlled trial, one was a case-control study, one was a prospective cohort study, and the remaining five were retrospective cohort studies.

Outcomes

The outcome was, in contrast with other races/ethnicities, Caucasians had the lowest risk of PPH, although they surprisingly had an increased risk of transfusion in comparison with Native Hawaiians and other Pacific Islanders (NHOPI) and Asians. Additionally, it was discovered that African Americans or African descendants had a higher risk of PPH but a lower risk of atonic PPH; they suffered greater consequences when they developed atonic PPH.

Discussion

In the United States and around the world, PPH is a primary cause of pregnancy-related morbidity and mortality [1,13]. While racial/ethnic inequalities in other obstetric outcomes have been thoroughly examined, there has been less detail documented on discrepancies in PPH [10,11]. The focus of this study was on the impact of race and ethnicity on PPH development in pregnant women which would better prepare clinicians for PPH prevention, early detection, and treatment.

Caucasians, Hispanics, Asians/Pacific Islanders, and Native Hawaiians

Compared to Caucasians, Hispanics, Asians, and NHOPI have an increased risk of atonic PPH. An increased probability of atonic PPH, in Hispanics, Asians, and Pacific Islanders, which required transfusion or hysterectomy was also reported [5,13]. In a secondary analysis, it was discovered that NHOPI and Asians had a greater rate of uterine atony although Caucasians had somewhat greater rates of transfusion than NHOPI and Asians, which was an unexpected finding [5].

Burmese nationality was not found to be a substantial risk factor for PPH; however, it was found to be a risk factor for severe PPH according to Thepampan et al. [18] in a nested case-control study examining a subpopulation of the Asian race at the Thai-Myanmar border hospital in Northern Thailand. This indicates that Burmese women have fewer hemorrhages than Thai women, but whenever they do, the hemorrhage is more extreme.

Caucasians, African, African American, and Middle Easterners

African Americans or African descendants had a considerably higher risk of PPH than Caucasians, with a decreased risk of atonic PPH, but a higher risk of atonic PPH requiring transfusion and hysterectomy [5,13]. This shows that African American women have fewer cases of atonic hemorrhage than Caucasian women. But when they experience atonic hemorrhage, it is much more severe, which can be explained in part by the greater proportion of comorbidities that increase the likelihood of atonic hemorrhage mandating transfusion or hysterectomy (e.g., fibroids, preeclampsia, and preexisting anemia) [5,9,13].

In secondary research of a randomized controlled trial of oxytocin for uterine atony prevention, Wetta et al. [16] discovered that Hispanic and non-Hispanic white ethnic origins were independent predictors for uterine atony requiring treatment and PPH when compared to African Americans [13,14].

Caucasians, Native Americans, and Middle Easterners

Native American women developed PPH at a far higher rate than all non-native women. Even though both groups had equal rates of chorioamnionitis, labor induction or augmentation, birth weight, placental anomalies, and parity, Native Americans had a considerably higher risk of uterine atony than the non-native group. PPH was much more common among Native American women when compared to white women. Native American ethnicity was discovered to be a significant determinant of PPH in the study [15].

In a German study, women from Asia, Africa, Latin America, and other nations had greater rates of hysterectomy. Asian women had a higher risk of hemorrhaging compared to German women, whereas women from the Middle East were less likely to develop hemorrhage. Inadequate prenatal care was connected to hysterectomy in Asian, African, Latin American, and other women, but not in Middle Eastern women [17].

Other biological predispositions such as coagulopathies, prostaglandin production, or tissue flexibility (degree of laceration) may also contribute to the increased PPH incidence in Asians/Pacific Islanders because postpartum hemostasis is interconnected and relies on the integration of various systemic factors [5]. Further research would be needed to (1) identify specific genetic factors in the races/ethnicities predisposing them to atony/suffering severe consequences from atony. (2) Determine whether races/ethnicities are independent risk variables while controlling for probable confounders.

Limitations and Strengths 

There are a few limitations associated with this study. The first is the scarcity of pertinent studies that are associated with the topic. Secondly, the study intentionally focused on more recent studies, specifically those published within the last 10 years . Thirdly, we restricted the study to materials published in the English language. The study also did not include animal studies and solely looked at human research. Finally, the study concentrated solely on open-access journals.

Talking about the strength of this research, one key strength is the intentionality in focusing on studies within the last 10 years only, to ensure relevance. Secondly, this study cuts across multiple ages, from the underaged (age 12) to middle age (>45 years old).

## Conclusions

This study aimed to discover the role played by race and ethnicity in influencing the development of PPH in child-bearing women. After conducting our research, we discovered that Caucasians have the lowest chance of acquiring PPH while having a higher likelihood of requiring blood transfusions. Similarly, African Americans or African descendants had a higher risk of getting PPH, as well as atonic PPH that necessitated transfusions or hysterectomy, but a reduced risk of developing atonic PPH. The study was aimed at (1) the selection of relevant evidence based on criteria and (2) a thorough analysis of the evidence. The goal of this study was to provide physicians and clinicians with evidence-based risk factors to consider while working with pregnant women. Case-control studies that examine race/ethnicity as a risk factor in the future should be explored by researchers; however, individuals with a prior history of PPH should be excluded from the control group to control for confounding.
